# Sarcopenia—The Impact on Physical Capacity of Liver Transplant Patients

**DOI:** 10.3390/life11080740

**Published:** 2021-07-24

**Authors:** Maciej Miarka, Krzysztof Gibiński, Maciej K. Janik, Renata Główczyńska, Krzysztof Zając, Ryszard Pacho, Joanna Raszeja-Wyszomirska

**Affiliations:** 1 Liver and Internal Medicine Unit, Department of General, Transplant and Liver Surgery, Medical University of Warsaw, 02-091 Warsaw, Poland; maciej.miarka@uckwum.pl (M.M.); maciej.janik@uckwum.pl (M.K.J.); 2 II Department of Clinical Radiology, Medical University of Warsaw, 02-091 Warsaw, Poland; krzysztof.gibinski@gmail.com (K.G.); ryszard.pacho@wum.edu.pl (R.P.); 3 I Department of Cardiology, Medical University of Warsaw, 02-091 Warsaw, Poland; renata.glowczynska@uckwum.pl; 4 Department of General, Transplant and Liver Surgery, Medical University of Warsaw, 02-091 Warsaw, Poland; krzysztof.zajactx@uckwum.pl; 5 Department of Radiology, Military Institute of Aviation Medicine, 02-091 Warsaw, Poland

**Keywords:** sarcopenia, end-stage liver disease, liver transplantation, cardio-pulmonary performance, L3SMI

## Abstract

Optimizing patients’ condition before liver transplantation (LT) could potentially improve survival of LT patients. We focused on sarcopenia, as a common factor in liver transplant candidates that can impact their cardiopulmonary performance at the point of listing, morbidity, and mortality after LT. We performed a single-center cohort study on 98 consecutive patients with liver cirrhosis who were transplanted between March 2015 and December 2017. The third lumbar vertebra skeletal muscle index (L3SMI) was calculated using CT imaging to distinguish sarcopenia at listing for LT. Data regarding liver function, body mass index (BMI), cardiac biomarkers, the peak oxygen uptake (VO2) and LT outcome were collected and correlated to L3SMI. For data analysis the Dell Statistica (Version 13. Dell Inc., Rondrock, TX, USA) was used. In total, 98 cirrhotic patients were included. Fifty-five (56.1%) patients, mostly males, had sarcopenia according to L3SMI, with the lowest L3SMI in males with alcohol-related liver disease. Lower L3SMI correlated with lower BMI, lower VO2 peak, and higher NTproBNP (all *p* < 0.001) and revealed an essential correlation with prolonged ICU stay (r = −0.21, *p* < 0.05). 33 patients were unable to perform cardio-pulmonary exercise test, mostly sarcopenic (67%), with more advanced liver insufficiency (assessed with CPC and MELD scores) and longer stay at ICU after LT (all *p* < 0.001). Sarcopenia was common among LT recipients. It was associated with inferior result in cardio-pulmonary performance before LT and prolonged ICU stay after grafting.

## 1. Introduction

Physical inactivity, sarcopenia, and frailty are highly prevalent, independent predictors of morbidity and mortality in patients with cirrhosis. In combination with advanced age, malnutrition, decreased hepatic protein synthesis, catabolism, hyperammonemia, increase in proinflammatory cytokines and low testosterone level, cardiac and skeletal muscle deconditioning results in reduced cardiac function and physical frailty as well as decreased skeletal muscle mass and strength [1].

Sarcopenia is defined by the triad of a generalized loss of skeletal muscle mass, strength, and function [2] and may be a marker of poor general status when predicting outcome in populations undergoing general surgery [3] and renal transplantation [4]. It seems to be a better indicator of malnutrition than serum albumin, weight, and body mass index (BMI), which may be modified by ascites in patients with liver cirrhosis. Nowadays, sarcopenia is recognized to be a highly prevalent and life-threatening complication of cirrhosis, irrespectively of the origin of the liver disease. Male-sex and decompensation of the liver function according to Child–Pugh class (CPC) C cirrhosis were independent predictors of sarcopenia what was an independent predictor of mortality [5]. Sarcopenia in liver transplant candidates was associated with increased waitlist mortality [5,6,7,8] and sepsis-related death on the waitlist [8]. Moreover, sarcopenia progresses during the transplant process and up to 1-year after surgery [9]. A recent meta-analysis showed the negative impact of sarcopenia on the results of liver transplantation (LT) [10]. Post-transplant outcomes associated with sarcopenia include prolonged hospital and intensive care unit (ICU) stay, increased risk of infection [11] and post-transplantation mortality [12,13,14,15,16,17].

Although the liver transplantation (LT) outcomes depend on recipients, donors and operative procedural characteristics, the shortage of donor livers increases the need to maximize the life-saving capacity of procured livers in terms of older candidates to surgery with more severe end-stage liver disease and greater comorbidities (e.g., metabolic syndrome). Additionally, cardiovascular events (CVE) contribute to excess serious complications and non-liver-related death after LT [18]. The goal of cardiovascular evaluation before LT is detection of severe cardio-pulmonary disease which makes LT futile. In general, cardiologic workup is focused on ischemic heart disease and its complications. However, there is a lack of routine dynamic evaluation of cardio-pulmonary performance of end-stage liver disease candidates to LT under controlled conditions of increased oxygen demand or hemodynamic instability. Cardio-pulmonary exercise test (CPET) might be a solution, because it simultaneously evaluates efficiency of circulatory, respiratory, and muscular systems, testing the ability of the cardio-respiratory systems to deliver oxygen to tissues under stress and provides an objective measure of functional capacity and impairment. A lower anaerobic threshold indicates lower aerobic fitness. Poor performance on CPET is associated with inferior postoperative outcomes and patients who are unable to complete CPET for some reason had the longest hospital stay and poorest survival [19]. Peak oxygen uptake (VO_2_ peak) below 60% predicted value was shown as a prognostic factor of 100-day liver transplant related mortality [20].

The impact of sarcopenia on cardio-pulmonary efficiency before LT and, further, morbidity and mortality after LT in order to prioritize patients by transplant survival benefit has not been studied as of date. However, it is rational to prioritize the patients to avoid short time futility, defined as posttransplant three-month mortality, as well as long-term survival below 50% at five years after LT.

Thus, we focused on sarcopenia in liver transplant recipients from a single liver transplant center and its impact on their cardio-pulmonary performance, and, further, morbidity and mortality after grafting.

## 2. Patients and Methods

### 2.1. Patients

It was a single center cohort study on 98 consecutive patients with liver cirrhosis who were transplanted between March 2015 and December 2017. Our cohort consisted of three subgroups of patients according to the etiology of liver cirrhosis: HCV, HCV complicated with hepatocellular carcinoma (HCC), and alcoholic liver disease (ALD), referring to Table 1. None of patients included in the study had medical history of myocardial infarction, stroke/transient ischemic attack or overt heart failure, neither abnormal right and left heart ventricular systolic and diastolic function in echocardiography. All the patients were successfully liver transplanted.

### 2.2. Study Design

#### 2.2.1. Definition of Sarcopenia

Although multiple studies have reported the impact of sarcopenia on outcomes in liver transplant candidates, no consistent definition of sarcopenia in this population exists. However, CT has been acknowledged as the gold standard for detection of low muscle mass by the European Working Group on Sarcopenia in Older People [2]. Thus, the recently published method by Carey et al. was applied [21]. The superior aspect of the L3 vertebral level was identified in CT scan and muscles at L3 were outlined. The cross-sectional area of the muscles was semi-automatically measured in cm^2^. After normalization of L3 skeletal muscle area to individual’s height, skeletal muscle index was calculated (L3SMI) with cutoffs of 50 cm^2^/m^2^ for men and 39 cm^2^/m^2^ for women [21].

#### 2.2.2. Structured Cardiologic Work-Up before LT

ECG, serum cardio-biomarkers (troponin I [TnI], N-terminal pro-B type natriuretic peptide [NTproBNP]), 24-h Holter-ECG, and contrast echocardiography were carried out in all liver transplant candidates. Additional testing, i.e., coronarography or CT scans with calcium score, heart MRI or SPECT were used when individually appropriate. Additionally, cardio-pulmonary exercise test (CPET) was intended to be performed in all individuals.

#### 2.2.3. Cardio-Pulmonary Exercise Test (CPET)

CPET, carried out by measuring maximal oxygen consumption at peak exercise (VO_2_ peak), provides a critical integrative approach of the aerobic capacity as well as a global assessment of the health status of an individual, physical fitness and physiological reserve. Due to assessment of sarcopenia and high percentage of ascites in study cohort, percent-predicted peak VO_2_ according to normative values were calculated without data about weight, by formula proposed by Jones et al. [22], as follows:

Male: VO_2_ (L/min) = 4.2 − (0.032 ∗ age),

Female: VO_2_ (L/min) = 2.6 − (0.014 ∗ age) with optimal cut-off predictive value of VO_2_ peak ≥ 80%, L/min.

### 2.3. Ethics

The study protocol was approved by the ethics committee of the Medical University of Warsaw (AKBE/119/2019) and conforms with the ethical guidelines of the 1975 Declaration of Helsinki (6th revision, 2008).

### 2.4. Statistical Analysis

Data is presented as mean and standard deviation (SD) or median with interquartile range (IQR), when appropriate. The Kolmogorov–Smirnoff normality test was used to examine the distribution of quantitative variables. Correlations were assessed by a nonparametric test—Spearman’s rank-correlation coefficient. The Mann–Whitney U-test, the Kruskal–Wallis test by ranks and Chi-square test for multidimensional contingency were used to check for differences between patient subgroups when appropriate. The factors associated with poor recipient survival or prolonged stay at ICU (defined as at least 6 days [23]) were analyzed on univariate and subsequent multivariable analysis (Cox proportional hazard and logistic regression analysis). Then, the Kaplan–Meier curves were created to examine patient survival by log-rank Cox analysis. For multiple comparisons, the Bonferroni correction were used. For data analysis, the Dell Statistica (Version 13. Dell Inc., USA) was used. A *p* value < 0.05 was considered statistically significant, except multiple comparisons when the Bonferroni correction was used.

## 3. Results

In total 98 consecutive liver cirrhotic and transplanted between Mar 2015 and Dec 2017 patients (F 23, M 75, mean age 55 ± 8 years) were enrolled into the study: 30 with HCV (F 11, M 19, mean age 55 ± 7 years), 26 with HCV complicated with HCC (F 6, M 20, mean age 58 ± 5 years) and 42 with ALD (F 6, M 36, mean age 54 ± 8 years). The mean BMI in the study group was 27 ± 4 kg/m^2^, MELD score 18 ± 9 points and most of patients were class B in CPC. However, in 19 (45%) individuals Child–Pugh class was C (mean 11 points). There were 15 HCV RNA negative patients of 30 (50%) with HCV cirrhosis of the liver at the moment of LT, but only 6 out of 26 (23%) in the group of HCV-HCC individuals.

In total there were 55 individuals (56%) with sarcopenia diagnosed by L3SMI: 44 (59%) men and 11 (48%) females. In the whole study group the mean L3SMI was 47 ± 10 cm^2^/m^2^: 41 ± 9 cm^2^/m^2^ in females and 49 ± 10 cm^2^/m^2^ in men. Patients with alcohol-related liver cirrhosis had the lowest L3SMI when compared to HCV and HCV-HCC subgroups (45 ± 10.3 cm^2^/m^2^ vs. 46.7 ± 10 cm^2^/m^2^ vs. 50 ± 8.9 cm^2^/m^2^; all *p* < 0.05).

Thirty-three individuals were unable to perform any exercise on the treadmill because of the immobilization and clinical frailty. Majority (67%) of patients unable to perform CPET were sarcopenic.

There was no significant difference between sarcopenic and non-sarcopenic patients in terms of liver failure scores, such as MELD and CPC as well as in the mortality after LT. However, there were substantial differences in MELD scores among subgroups, with the highest values in HCV individuals (23 ± 8.3 points), followed with ALD cirrhotic patients (18.4 ± points) and HCV-HCC transplanted individuals (12.5 ± 6.5 points) (all *p* < 0.001). Clinical data regarding sarcopenia was summarized in Table 1. Lower L3SMI correlated with lower BMI, lower VO_2_ peak, higher NTproBNP and correlated with prolonged ICU stay (r = −0.21, *p* < 0.05). Thus, sarcopenic patients presented reduced cardio-pulmonary performance and were more prone to longer stay at the ICU (Table 2).

Moreover, there were substantial differences between patients who performed CPET and individuals unable to perform any exercise in terms of CPC (*p* < 0.05) and MELD, L3SMI, NTproBNP, as well as prolonged ICU stay (all *p* < 0.01).

**Figure 1:**
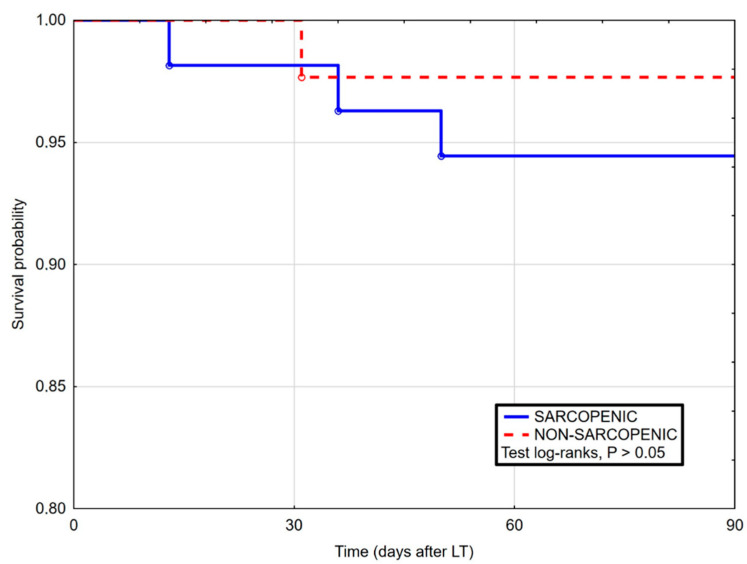
Kaplan–Maier curves for survival after liver transplantation in sarcopenic and non-sarcopenic patients in 90 days follow-up. Log-rank Cox (HR 2.46, 95% CI 0.34–17.65, *p* = 0.420).

**Figure 2:**
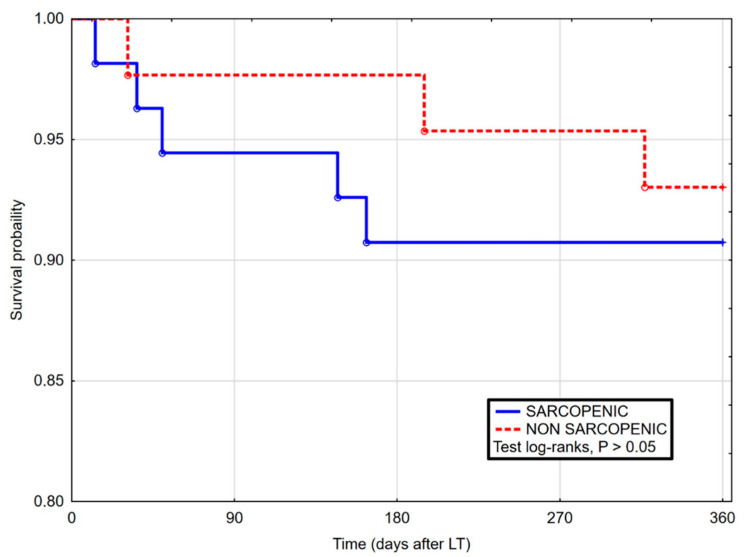
Kaplan–Maier curves for survival after liver transplantation in sarcopenic and non-sarcopenic patients in 360 days follow-up. Log-rank Cox (HR 1.05, 95% CI 0.28–3.90, *p* = 0.943).

**Figure 3:**
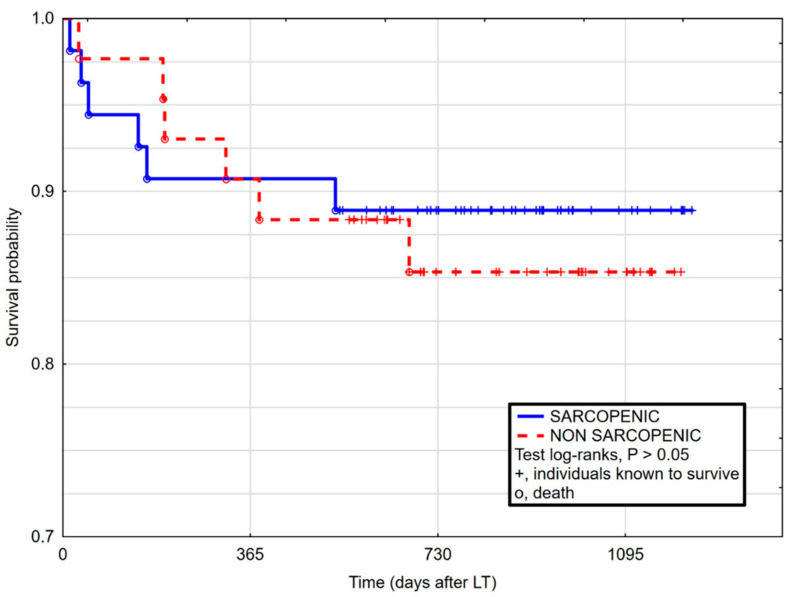
Kaplan–Maier curves for survival after liver transplantation in sarcopenic and non-sarcopenic patients in full follow-up period. Log-rank Cox (HR 0.82, 95% CI 0.26–2.56, *p* = 0.734).

In comparison, in the Kaplan–Meier curves for patients with and without sarcopenia, no significant differences were reported in terms of survival in 90-day post-LT (Figure 1), as well as 1 year (Figure 2) and whole follow-up period (up to 1224 days after surgery) (Figure 3).

## 4. Discussion

In this paper, authors pointed out the high number of sarcopenic patients awaiting liver transplantation with correlation between sarcopenia and prolonged ICU stay after LT but not with mortality. More than half of evaluated individuals in analyzed cohort presented sarcopenia. Sarcopenia seemed to be a derivative of alcoholic cirrhosis and more advanced liver disease; both were true for males with end-stage liver disease, suggesting sex differences in this term. In ALD patients both advanced liver disease and alcohol directly contribute to sarcopenia with myosteatosis reaching 68% in sarcopenic patients [24]. Alcohol impairs skeletal muscle mitochondrial function and protein synthesis [25], leads to hypogonadism and its effect on muscle [26,27] It inhibits mammalian target of rapamycin complex 1 which role is suppressing autophagy and stimulating protein synthesis [28]. Moreover, end-stage liver disease of alcoholic origin was associated with loss of muscle and fat mass as described by Benjamin et al. [29]. This alteration in body composition accompanied the progression from compensated to decompensated liver cirrhosis. [29]. There was no correlation between sarcopenia and advanced liver impairment in the present study. This finding was in line with data of Kang S.H. et al., who found that MELD was not an independent predictor of mortality in severe sarcopenia in the study with 452 cirrhotic patients [30] and pointed out the role of novel MELD-sarcopenia score [31]. On the other hand, the additional value of MELD-sarcopenia index in allocation of organ system was questioned by van Vugt et al. [32].

The universal structured cardiologic protocol applied in liver transplant centers is dedicated mainly for detection of coronary artery disease and its complications. Of note, dobutamine-stress echo is not a standard of cardiac evaluation to LT across the centers. Disadvantages of such strategy is associated with the lack of assessment of heart function during surgery with increased oxygen demand as well as in hemodynamic instability. The novel aspect of presented analysis is the impact of sarcopenia on worse cardio-pulmonary performance, i.e., lower VO_2_ peak and higher NTproBNP before surgery. These results corroborate previously published data regarding the impact of aerobic capacity on LT prognosis [20,33]. Reduced VO_2_ peak has been independently associated with 100-day mortality following LT [20]. Liver transplant candidates with greater VO_2_ peak experienced significantly better 12-month survival after surgery [34]. Moreover, VO_2_ at ventilatory threshold was the only predictive factor of mortality after LT [33]. A reduction in aerobic power or aerobic endurance correlated with increased mortality [34,35], and might be a more sensitive predictor of mortality than sarcopenia alone, pointed rather to the muscle function, i.e., chronic immobilization, than mass [36].

What is more, we also found higher levels of plasma NTproBNP in sarcopenic individuals, supporting the thesis of impaired heart function in terms of reduced muscle mass and strength, as well as mirrored data regarding the reverse impact of higher body mass index on this cardiac biomarker [37,38]. NTproBNP is an independent risk factor of all-cause mortality in patients without heart failure [39]. Moreover, newly established cardiac cachexia of multifactorial origin—including endotoxemia, chronic inflammation, long-term neurohormonal overactivation, and upregulation of the transcription the proteolytic ubiquitin–proteasome pathway components—has been described, with only NTproBNP recommended to diagnose this condition as of the date [40].

The results of the present study regarding the risk of prolonged ICU stay in sarcopenic patients are in keeping with previously published data. DiMartini et al. found that sarcopenic patients of both genders were at risk of prolonged ICU stay after liver transplantation, total hospital stayand days of intubation, however, with modest effect in female patients [12]. The possible explanation of these findings might be lower respiratory quotient in sarcopenic individuals [41]. Neviere et al. showed that survivors after LT at one-year follow-up had higher VO_2_ peak when compared to non-survivors [42] and lower VO_2_ peak was consisted with longer ICU stay [43]. Moreover, some new data points to the impact of sarcopenia on reduced left heart chamber with occult heart failure (with preserved ejection fraction in basic condition) [44] as well as respiratory muscle and diaphragmatic sarcopenia with their weakness [45,46].

Thus, the novel aspect of this analysis is the impact of sarcopenia on worse cardiac performance before surgery with the higher risk of prolonged ICU stay with its further complications after LT in sarcopenic individuals unable to perform any exercise on the treadmill. The population of LT candidates unable to perform an objective CPET should be of interest in planning early recognition, testing and management of sarcopenia and muscle function. As of date, there were no studies analyzing sarcopenia regarding an individual’s cardiopulmonary performance [47].

In the study of Kalafateli et al. with 232 consecutive liver transplant recipients, prolonged ICU stay was derivative of severe malnutrition and patients with sarcopenia receiving suboptimal graft had longer ICU and hospital stay with higher incidence of infection and higher one-year mortality after LT [15]. However, the population of transplant candidates unable to performed CPET were underscored in the present study in respect of their maximal exercise capacity as well as their heart function under stress of increased oxygen demand during surgery and intravascular volume changes. Thus, the structured cardiac pre-transplant protocol applied in our center to evaluate the cardio-vascular risk in liver graft recipients was unable to identify individuals with lower benefit of LT. There were also some other limitations of the present study. We did not prove increased mortality in sarcopenic liver graft recipients what is mentioned in literature - recently published meta-analysis of eleven studies clearly pointed to the increased mortality in transplanted individuals with low muscle mass [10]. Thus, the negative result of the present study might reflect underestimation resulting from the number of participants. On the assumption of *p* value < 0.05, accepting statistical level error 0.02 (2%), the study cohort should consist of 142 patients. Furthermore, we had no data regarding the role of ammonia and testosterone levels as well as a nutritional treatment—i.e., branched-chain amino acids (BCAA) enriched diet in therapy of sarcopenic individuals during run-up to liver transplantation. Although, as of date, there is no convincing data showing the benefit of diet and testosterone supplementation in end-stage liver disease [48]. Moreover, the role of physical activity were studied in patients with compensated cirrhosis, usually in CPC class A, although not in candidates to LT with ESLD [1,49]. The impact of anti-viral therapy with novel DAA drugs on patients’ general condition was also not studied as of date. On the other hand, structured cardiologic protocol was dedicated mainly for the detection of coronary artery disease and its complications. Disadvantages of such a strategy were associated with evaluation of coronary arteries at rest with the lack of assessment of the heart function as in surgery, with increased oxygen demand as well as in hemodynamic instability. The impact of sarcopenia on cardiovascular morbidity and mortality after LT in respect of prioritizing patients by transplant benefit in terms of morbidity was not clearly demonstrated in this present study.

In conclusion, the results of the present study are in line with the previously published data linking sarcopenic patients with worse cardiopulmonary assessment result and prolonged ICU stay after LT. The urgent need for early identification and appropriate cardiac testing of sarcopenic LT candidates exists. CPET might be a solution, however only in individuals capable of effort on treadmill. Therefore, there is an unresolved problem with evaluation of immobilized or frailty liver transplant candidates. The population of LT candidates unable to perform an objective CPET should be of interest in planning early recognition, testing, and management of sarcopenia and muscle function.

## Figures and Tables

**Table 1:** Comparison between patient with and without sarcopenia, defined by L3SMI.

	Sarcopenic (*n* = 55)	Non-Sarcopenic (*n* = 43)	*p* Value
Age	55 (9)	57 (9)	0.244
Gender (female)	20%	28%	0.364
Etiology HCV HCC ALD	29% 20% 51%	32.5% 35% 32.5%	0.716 0.100 0.071
BMI (kg/m^2^)	24.8 (5)	29.3 (5)	<0.001
MELD (points)	18 (14)	16 (11)	0.161
CPC (points)	10 (3)	9 (4)	0.289
VO_2_ Peak (J. eq.)	63.2 (33)	79.3 (29)	0.064
TnI	0.009 (0.02)	0.010 (0.03)	0.988
NT-proBNP	176 (263)	74 (79)	0.043

Abbreviations: ALD, alcohol liver disease; BMI, Body Mass Index; CPC, Child–Pugh Class; HCC, hepatocellular carcinoma; MELD, Model of End Stage Liver Disease; NT-proBNP, N-terminal pro-brain natriuretic peptide; TnI, troponin I. The data are presented as median and IQR or percent of the subset, when indicated. The U-Mann test was performed to compare the subgroups. *p* value < 0.05 considered significant.

**Table 2:** Correlations between sarcopenia, cardiac markers, and clinical variables.

	L3 SMI	VO_2_ Peak (J. Eq.)	TnI	NT-proBNP
**L3SMI**		0.592 **	−0.095	−0.531 **
**VO_2_ Peak (J. eq.)**	0.592 **		−0.098	−0.354
**TnI**	−0.095	−0.098		0.107
**NT-proBNP**	−0.531 **	−0.354	0.107	
**BMI**	0.473 **	0.485 **	0.197	−0.056
**MELD**	−0.159	−0.151	0.380 *	0.266
**CPC**	−0.159	−0.142	0.344 *	0.088
**ICU Stay**	−0.208 *	−0.217	0.087	0.358 *

Abbreviations: BMI, Body Mass Index; CPC, Child–Pugh Class; L3SMI, third lumbar vertebra skeletal muscle index; MELD, Model of End Stage Liver Disease; NT-proBNP, N-terminal pro-brain natriuretic peptide; TnI, troponin I. The Spearman test was performed. *p* value < 0.05 considered significant. The data are presented as rho. * *p* < 0.05; ** *p* < 0.01.

## Data Availability

Data described in the manuscript, code book, and analytic code will be made available upon request pending on application and approval.

## Abbreviations

ALD, alcoholic liver disease; BMI, Body Mass Index; CPC; Child–Pugh class; CPET, cardio-pulmonary exercise test; CVE, cardio-vascular event; ESLD, end-stage liver disease; F, female; HCC, hepatocellular carcinoma; HCV, hepatitis C virus; ICU, Intensive Care Unit; MELD, Model of End-Stage Liver Disease; L3SMI, the third lumbar vertebra skeletal muscle index; LT, liver transplantation; M, male; MELD, Model for End Stage Liver Disease; NTproBNP, N-terminal pro-B type brain natriuretic peptide; TnI, troponin I; VO_2_peak, peak oxygen uptake
